# Children’s description of pain through drawings and dialogs: A concept analysis

**DOI:** 10.1002/nop2.211

**Published:** 2018-10-14

**Authors:** Fatemeh Ebrahimpour, Shahzad Pashaeypoor, Waliu Jawula Salisu, Mohammad Ali Cheraghi, Akram Sadat Sadat Hosseini

**Affiliations:** ^1^ School of Nursing & Midwifery Tehran University of Medical Sciences Tehran Iran

**Keywords:** children, concept analysis, nurses, nursing, paediatrics, pain

## Abstract

**Aim:**

To present a concept analysis of pain in children's drawings and dialogs.

**Introduction:**

The complexity and subjectivity of the concept of pain in children remain ambiguous. As a result, children are exposed to inappropriate diagnosis and inadequate treatment. Children can describe or draw their painful experiences. Analysing the concept of pain based on children's experiences can help identify, assess and properly manage and treat pain in children.

**Design:**

Concept analysis.

**Methods:**

Walker and Avant's framework for concept analysis was used in this current study.

**Results:**

Major aspects of pain revealed in this concept analysis are affected by children's different concerns about pain. The description of pain in children with chronic diseases or chronic pain is completely different from that in healthy children. Children perceive pain to be internal, external and emotional. Pain in children is associated with poor psychological and emotional conditions, which add new features and aspects to the concept of pain. Children's descriptions and drawings of pain indicate different concepts of pain in their minds. From the perspective of children, pain has an identity that is formed based on reality.

**Conclusion:**

When developing pain evaluation tools, it is necessary to address the characteristics of pain. In the case of chronic pain, emotional effects of pain on children's psyche need extra attention. Child‐based pain management guidelines can then be formulated with the results of relevant concept analyses. Pain assessment is a major part of pain management in children. By considering the characteristics of the concept of pain, the efficiency and usefulness of developed tools can be enhanced to create advancement in paediatric pain management.

## 1 INTRODUCTION

Every living being is familiar with the phenomenon of pain and has similarly lived through it (Wells, Pasero, & McCaffery, 2008). Irrespective of this universality of pain, it is yet a complex and multidimensional experience which is hard to clearly define (Montes‐Sandoval, 1999; WHO, 2012) and even more troubling when trying to understand what pain is among children. Cheng, Foster, and Hester (2003) and Cheng, Foster, and Huang (2003) suggest that pain is a distressful and an unpleasant feeling which when left unrelieved, could affect the quality of life of an individual. Other studies found that chronic pain in children leads to increased levels of anxiety (Smith, Sumar, & Dixon, 2014). Despite this, a study by Friedrichsdorf, et al. (2015) revealed that pain in children often goes unnoticed and undertreated. Just like adults, children are equally endowed with the ability to perceive pain from the early stage of their prenatal life till the development of the nervous system which is associated with the sense of pain (Sekulic, et al., 2016). However, the major challenge is how children could effectively communicate their pain experiences to caregivers and significant others for them to be appropriately understood and cared for.

## 2 BACKGROUND

Children can experience different types of pain—acute, recurrent and chronic (IASP, 2005). The World Health Organization (WHO) has correspondingly paid special attention to the experience of pain in children because they may be neglected due to their inability to communicate verbally or to express their pain clearly (WHO, 2012). In this respect, the children's age and stage of development can have effects on their pain perceptions and descriptions (IASP, 2005). Children are at the risk of being misunderstood by others in terms of their pain and also falsely diagnosed and treated (Mathews, 2011). Pain is not visible, and its subjective nature is the result of personal experiences (Lewandowski, Good, & Draucker, 2005).

In 1971, children's subjective pain perceptions were studied for the first time, and the results revealed that children could describe the nature of their pain verbally or through drawings (Schultz, 1971). Recent clinical research studies have made major contributions to expand this body of knowledge of pain in children, clarifying how children cope with acute and chronic pain ( Meldrum, Tsao, & Zeltzer, 2009; Borghi, et al., 2014; Pope, Tallon, McConigley, & Wilson, 2015; Smith, et al., 2014), cognitive and emotional aspects of pain (Kortesluoma, Nikkonen, & Serlo, 2008; Kortesluoma, Punamäki, & Nikkonen, 2008), describing pain experiences with drawings (Jongudomkarn, Aungsupakorn, & Camfield, 2006; Unruh, McGrath, Cunningham, & Humphreys, 1983) and children's perceptions of pain (Chaves, et al., 2013). Literature keeps growing in this subject area covering different aspects of pain in children (Kortesluoma & Nikkonen, 2006; Meldrum, et al., 2009; Neuman, 1996; Pölkki, Pietilä, & Vehviläinen‐Julkunen, 2003; Pölkki, Rissanen, & Pietilä, 1997). However, with these advances, the concept of pain in children still remains ambiguous. For example, researchers agree that children's drawings give an important guide about their experiences of pain (Kortesluoma, Nikkonen, et al., 2008; Kortesluoma, Punamäki, et al., 2008). Yet, this literature is scanty and does not give clear descriptions and interpretations of drawings children use to represent pain, leaving this area less explored.

A clear understanding of the complexity and subjectivity of the concept of pain in children can enhance diagnosis and improve treatment. Thus, a concept analysis is necessary to clarify and make such ambiguities more understandable. Concept analyses have been used widely in studies of quality of life (Meeberg, 1993), trust (Gonzalez, 2017), pain (Cheng, Foster, & Hester, 2003; Cheng, Foster, & Huang, 2003), risk (Shattell, 2004), confidence/self‐confidence (Perry, 2011), resilience (Garcia‐Dia, DiNapoli, Garcia‐Ona, Jakubowski, & O'Flaherty, 2013) and more. Further, numerous studies have been conducted to evaluate children's experiences and descriptions of pain (Jongudomkarn, et al., 2006; Meldrum, et al., 2009; Pölkki, et al., 2003; Savedra, Tesler, Holzemer, Wilkie, & Ward, 1982; Schultz, 1971; Stefanatou & Bowler, 1997). However, there is no complete and clear definition of pain in children, and the pain in children's drawings and dialogues has not been widely explored. Therefore, the aim of this study was to clarify the concept of pain using concept analysis approach with emphasis on how children perceive and describe pain with drawings.

## 3 METHODS

We explored the concept of pain from children's perspectives and determined its characteristics and components using Walker and Avant (2011) method of concept analysis (Walker & Avant, 2011). This approach is better suited for nurse's needs and allows authors the liberty to iteratively use its outlined steps (Meleis, 2012). Also, the current text available about the concept to be explored informed the choice of this methodology. It is considered to be the most frequently used method of concept analysis in the literature and consists of eight steps. To identify both implicit and explicit uses of the concept, the authors used dictionaries, colleagues and literature available. The literature review provides the evidence base for analysis of the concept and validates the choices of the defining attributes. Furthermore, an in‐depth literature review is intrinsic to concept analysis, and a critical analysis of the literature can represent the entire approach to concept analysis. To obtain substantial data necessary for clarification and understanding of the concept, we conducted a literature search in scientific databases such as PubMed, Google Scholar, EMBASE, ProQuest and Web of Science. Using search terms such as “Pain,” “Children,” “Pediatrics,” “Drawing” and “Dialogs” without limits to the year of publications, a total of 256 articles were retrieved. For the review and critical analysis of the concept, we then selected and included 23 relevant studies after screening titles and abstracts. Also, free Internet searches were conducted when necessary to retrieve relevant information. Articles that assessed pain among children with a focus on drawings, dialogs or interpretations of descriptions of pain were those that met the inclusion criteria. The analysis was conducted following the eight‐step approach proposed by Walker and Avant (2011) as shown in Table 1.

**Table 1:** Walker and Avant’s (2011) proposed steps in conducting concept analysis

Steps		Approach
1	Selecting the concept	As an initial step, we focused on the concept of pain among children and how children represent pain through drawings and dialogs
2	Determine the aims or purposes of the analysis	We clarified our aims of conducting a concept analysis in this step
3	Identify all uses of the concept that you can discover	Using dictionaries, and other available Internet sources, we identified regular usage of the concept of pain
4	Determine the defining attributes	We classified descriptions based on their applications and identified prevailing differences
5	Identify a model case	Examples of the concept application which reveal its definitive characteristics were provided at this stage.
6	Identify borderline, related, contrary, invented and illegitimate cases	This stage consists of identifying some cases: Borderline cases: Cases that do not give clear descriptive definitions of the concept Related cases: Identifying cases that are closely related to the concept Contrary cases: These are cases that are clearly not related to the concept of interest Invented cases: Crated cases with minimal relatedness to the concept Illegitimate cases: Wrong and non‐representing cases. This step (6), however, did not occur because the authors found it less relevant to the goals of this current study
7	Identify antecedents and consequences	For a concept to occur, there must be certain conditions and events present, and as well some consequences. In this stage, we identified these conditions, events and consequences
8	Define empirical referents	As a final stage, we tried to answer questions such as how to measure the concept of pain in a practical environment

### 3.1 Selecting the concept

Pain is a global health problem (Goldberg & McGee, 2011), and its significance is to the extent that it has been considered the fifth sign of human life (Mularski, et al., 2006). However, the concept of pain in children has been unclear for many researchers and the medical community; and no definite, accurate and practical definition has so far been proposed. Since children have different perceptions of pain in various social and environmental contexts, aggregation of commonalities can lead to a comprehensive understanding of pain by nurses and doctors.

### 3.2 Determine the aims or purposes of analysis

The purpose of this study was to clarify the concept of pain in children by identifying key elements, distinctive characteristics, and antecedents and consequences.

### 3.3 Identify all uses of the concept that you can discover

The etymology of pain is from the Latin word *poena*, which translates as punishment, penalty, retribution and indemnification. From Greek, its *poine*, which is retribution, penalty, quit‐money for spilled blood, and Old French, *peine* which also is a difficulty, woe, suffering, punishment, hell's torments. In the late 13th century, two practical meanings were raised for the term *pain*: (a) punishment: especially for a crime and (b) condition on feels when hurt, opposite of pleasure (Dictionary, 2016).

In Webster's Medical Dictionary, pain is defined as a state of physical, emotional and psychological discomfort. Thus, pain may be local or general and it can also have physical and psychological consequences. Generally, pain is an answer to avoid or eliminate the factor that caused it. Additionally, pain is usually limited to a physical harm associated with a physical disorder such as a disease or an injury (Merriam‐Webster, 2016). In most definitions, pain is described as subjective and highlighted as a reaction to discomfort in response to an acute or potentially abnormal tissue damage that is received intuitively and perceptibly and also can lead to changes in the body, emotions and psyche (Bushnell, Čeko, & Low, 2013; Crofford, 2015; Merskey & Bogduk, 1994).

### 3.4 Determine the defining attributes

The characteristics of the concept of pain were extracted from children's perspectives based on the main findings of related studies on children's experiences and descriptions of pain and then illustrated in Table 2. The main characteristics of the concept of pain from the children's perspectives included a source of pain, pain as a physical harm, pain as a negative emotion, pain as a concern and pain descriptors.

**Table 2:** Concept of pain in children

Study	Results (current and past experiences of pain in children)	Pain characteristics—from the perspective of children
Savedra et al. (1982) Meldrum et al. (2009) Bienvenu et al. (2011) Hermann and Blanchard (2002)	The causes of pain: external, (falling, falling off), internal (illness, surgery) and psychology (when my mother leaves me) Descriptive words of pain: sore, ache, discomfort, cuts, insect bite, injury, misery, slashing, intolerable pressure, terrible Illness and illness, hot, itching, shooting, cold, warm, bitterness, bothering, boredom and cruelty Descriptive words of pain in hospitalized children: pain (sore, ache), uncomfortable situations, misery, intolerable procedures, terrible drugs and illness A child's feelings about pain: sick and abdominal discomfort, fear. This condition will never be lost, and this pain and state will not be separated from me. Feeling of crying but not crying. Feeling of crying and crying. Damage and hurt by someone or something. Being nervous and angry The colour of pain: black and red What is good for pain? Half answered; there is nothing good about pain. Some children said I do not know. Some answered: pain makes experiences for us. Pain means you have done bad deeds and have been punished. If it does not, we will not notice that our hands are burnt. Pain tells you that something is wrong What lowers your pain? Medicinal and non‐medicinal (inform others, rest and eat and drinking, warming or cooling the place, hugging and caressing, etc.)	The source of pain can be external and internal Pain is a physical harm or a mental discomfort Pain refers to a feeling of misery, trouble and discomfort Pain is an ailment and an illness Pain is frightening Pain can occur in a specific part of the body Pain is inherent in children and it is not separated from them Pain contains emotional reactions Pain is dark Pain is a sign of danger and precaution Pain becomes visible, and it is not hidden Pain is considered as a punishment for a child's wrong‐doing Pain should be relieved Pain is a warning sign of a bad event The bad aspects of pain outweigh the good ones Some children are not aware of the good aspects of pain Experiences and descriptions of pain in healthy children are different from sick children There is a need to get help from others to relieve pain
Unruh et al. (1983) Jongudomkarn et al. (2006) Kashikar‐Zuck et al. (2005) Robins et al. (2005)	Pain in children's drawing: Pain has been painted with some tools (two large pieces of a stick at the head that presses the head from both sides) The location of the pain was shown An abstract image of pain was drawn like a light arc A physiological representation of the pain, which traced the pathways of pain in the brain The pain is drawn in the form of humans that has straight hair up Painting a child with pain: A child who cries A child who is relieved of pain A child who had a migraine had painted several hammers around his head The child painted himself with the part of his body that has pain The black and red colours are the most colours used by children in the painting of pain	Pain is dark The source of pain is external Pain is a sign of danger and precaution Pain is a physiological process Pain causes distress and suffering Pain is not visible by itself, but a child affected with pain is different from other children Pain occurs in a specific part of the body Children are able to show the location of pain Pain can be countered Pain is annoying and causes crying Pain has a certain cause
Gaffney and Dunne (1986) Stefanatou and Bowler (1997) Merskey and Bogduk (1994) Bushnell et al. (2013)	Pain is a thing/something Pain is what's in my stomach Pain is what's in my body Pain is an unpleasant physical state Pain is harm Pain associated with illness or trauma Pain is one thing when you fall or become ill The pain is a sore feeling (pain) Pain is a sense of injury Pain is an emotion when we get sick Pain is an injury to the leg Pain is mental and physical suffering, physical and mental harm, depression and anxiety	Pain is inherent in children, and it is not separated from them Pain occurs in a specific part of the body Pain is a sense Pain is a physiological process Pain is a physical and mental discomfort Pain is anxiety‐provoking Pain is a symptom of harm and injury Pain is an illness and a trauma The source of pain is external Pain has an emotional and cognitive effect
Crow (1993) Jongudomkarn et al. (2006) Perquin, et al. (2003)	What is a pain? Pain is something that happens to my leg What is your experience of pain? Falling from a bicycle, injuries, illnesses, psychological and emotional pain What do you feel if you have pain? The pain feels bad, crying, sadness, hurt. I wish it would get away from me. The pain is like you're ready to die How is the pain caused? I do not know, falling, cutting, something weird happens in our body What does pain mean? I do not know, damage and injury, the pain is caused when you hurt your heart What are the similar words of pain? Suffering, anger, needle, pain, burning Where is the pain place? In part of the body How does the pain get better? Sleep, rest, stroking, kissing, playing, talking, watching TV Why is pain bad? Because it's harm, we cannot do anything else; we cannot do what we like Is there a good pain too? I do not know, no good pain	Pain represents harm and injury Pain can be countered Pain is a physical harm or a mental discomfort Experience of pain comes from everyday life events and medical, psychological and emotional ones Pain is an emotional sense (sense of crying) and a non‐emotional one (sense of being hurt) Having no pain is a desire for children Pain is deadly Pain occurs because of strange events in the body or someone and something external hurting the body Pain disrupts the course of life There is no good pain Pain is bad Pain is suffering There should be no pain
Neuman (1996) Kortesluoma, Punamäki, et al. (2008) Bienvenu et al. (2011) Crofford (2015)	Causes of pain: falling, crashes, injections, surgery Emotions related to pain and injury: hot, cold, dizziness, hunger, weakness, burning, itching, pain, discomfort, sadness, fear, anger, crap, insect sting Factors that relief pain: play, watch TV, cartoons, wash your injury site, cool down your injury site, rest and eat, medication, hug, touch and massage The contents of pain: a person and a body part, medical equipment and procedures, emotions, abstract presentations	Pain is an unpleasant feeling to describe Pain can be relieved Medication is not the only cure for pain There are two types of pain: accident and medicine Pain has a cognitive and an emotional effect
Cheng (2002) Cheng, Foster, and Hester (2003)	Pain is a feeling of discomfort, bad, uncomfortable, signs of illness, dying, feeling of fear, feeling bad A child who has pain is crying, not laughing, screaming, upset, someone who has been injured (falling, crashes, surgery, injections, toothache, hospitalization) If a child has pain due to surgery, this pain comes out of her brain and mind, but when she is alone and unloved, this pain comes out of her heart The pain of knowing one thing is wrong The most commonly used word for describing your pain is voice (ooh, waaa, shhh. etc.), feeling bad, discomfort and pain Pain is upset, do not play, feel bored, injections Pain in children's drawing: Showing the location of pain, describing and writing the words associated with pain along with the painting (I'm sad, I'm going to die soon, why me, help, pain), showing emotional pain (pain in the heart) Showing pain like a cloud "It's dark that shoots the sun, pain makes me feel bad, like a rainy day that does not have an umbrella with you; I feel the whole of my body is crying"	Pain causes behavioural reactions Pain is an unknown feeling There is a need to get help for pain control Pain is a physical harm or a mental discomfort Pain is hurt Pain occurs in a specific part of the body Pain has good aspects such as more attention to children and bad aspects like making changes in the normal course of life Children often draw pain in the form of complications and feelings associated with it Words, sounds, sentences, behaviours and drawings are among the manifestations of pain in children
Visram (2009) Cheng, Foster, and Hester (2003) Degotardi, et al. (2006) Tsao et al. (2012)	Pain is distress, uncomfortable, cramping, sore, ache, irritation and annoyance Pain is that someone is beating me, a knife in me, I feel my eyes are on my knee and somebody presses me in my eyes and makes a hole, it feels like I'm dying	Chronic pain is an annoyance Chronic pain tolerance is difficult Pain puts the whole body under pressure Pain is a sense of dying Pain is extrapolated in a child's mind Chronic pain is not well controlled
Borghi et al. (2014) Perquin et al. (2003)	"It hurts when the bandage is removed, it is a pain that hurts, it is a pain that pulls, it makes me cry and it is tiring. If I had to rate pain, from zero to ten, I would say that my pain is a 10!' "I recently had a tumor removed; I felt pain, a lot of pain, a strong pain that throbbed! In addition to that, I have headaches, which makes me crazy." Using many alternatives for managing pain Seeking a life that is closer to normality, despite pain and disease	Children suffering from chronic pain try to make use of all methods to return to normal life Return to normal life is the most important desire in children Chronic pains are severe, exhausting, frustrating and gloomy for children

#### 3.4.1 Source of pain

One of the characteristics of the concept of pain in children is the source or cause of pain. Considering the source of pain, all children assume it as an unpleasant thing hurting them elsewhere. Children also know the sources of pain as internal, external or emotional. The external pain has a distinguished source, it can be seen, and it can be mostly perceived by children (Bienvenu, Jacquet, Michelutti, & Wood, 2011). In this respect, children assume the sources of external pain in natural events such as falling down, cuts, burns or even treatment procedures performed by medical teams. It seems that such pain is more justifiable for children because they see and identify the cause; however, they do not like it and they want it to be quickly resolved. Children also perceive pain and can describe it or actually draw and paint it (Jongudomkarn, et al., 2006; Meldrum, et al., 2009; Stefanatou & Bowler, 1997).

Nevertheless, the source of internal pain is often unclear to children (most especially those with cognitive impairments) since they do not know its actual cause, progress, or sequelae (Mazur, Winnicki, & Szczepański, 2013). Children primarily attribute the cause of internal pains to diseases. Likewise, children cannot perceive the cause of disease and how the disease brings about pain (Kashikar‐Zuck, Swain, Jones, & Graham, 2005). Unlike external pain, internal pain does not have a visible cause in children, but they know that something bad has happened and pain becomes their informants. However, they do not know exactly what the pain is about. They are worried and they would like to relinquish the situation by any means and continue their lives normally (Jongudomkarn, et al., 2006).

The source of children's emotional pain is in the heart. They do not have the ability to express the mental states of emotional events accurately such as loss of their parents or friends and they often express it in the form of pain (Meldrum, et al., 2009; Savedra, et al., 1982).

#### 3.4.2 Pain as a physical harm

One of the main characteristics of the concept of pain that is apparent in children's dialogs and drawings is the synonymy of the concept of pain with a physical harm or a physical discomfort (Bienvenu, et al., 2011). In other words, the senses of pain that are pictured in the minds of children show someone with a physical harm which is mainly related to the external source of pain, and it refers to diseases in some cases. Pain in children's drawings or speech is the same place that is hurt on the body and they may colour it (Kortesluoma, Nikkonen, et al., 2008; Kortesluoma, Punamäki, et al., 2008).

#### 3.4.3 Pain as a negative feeling

Children find pain as a kind of feeling that is not pleasant. From children's perspectives, pain is something that causes crying and discomfort (Meldrum, et al., 2009). Pain as a feeling has the following characteristics:

- Its main nature is unpleasant and negative (Crofford, 2015).
- It is placed in a spectrum of mild to severe intensity; for example, from the feeling of pinches to senses of dying and pain intolerance (Bienvenu, et al., 2011).
- It can be associated with all three sources of external, internal and emotional pain (Bushnell, et al., 2013; Crofford, 2015).
- It includes two types: (a) emotions related to the stimulation of the body's peripheral sensory nerves such as a sense of warmth or cold, pressure, needle punching; and (b) emotional feelings such as sadness, fear, anger, discomfort, misery and crying (Bienvenu, et al., 2011; Hermann & Blanchard, 2002; Meldrum, et al., 2009; Robins, Smith, Glutting, & Bishop, 2005).

#### 3.4.4 Pain as a concern

Pain for children suggests a physical harm or a negative feeling. These conditions are also accompanied by worries and concerns, and children may find pain as their main cause (Bienvenu, et al., 2011). These concerns form up important aspects of the concept of pain in children including:

- Children perceive pain as a sign of an injury or a problem in the body. This is a concerning issue for them especially in children of preschool age who are afraid of being injured. So, pain for them is manifested in the senses of being unwell, losing the body or dying (Degotardi, et al., 2006; Visram, 2009).
- The presence of pain associated with any source of pain can cause restricted movements or activities in children. Thus, children get worried and feel sad about the pain preventing them from playing or going to school. Accordingly, pain is conceptualized for them in the sense of limiting or inhibiting the activities and the desired actions (Perquin, et al., 2003).
- Sometimes children experience hospitalization or receive treatment procedures for their pain relief. These stressful events can also have an effect on the concept of pain in children, and they may consequently interpret pain in the senses of hospitalization and injections. Likewise, this concept reflects the concern and the fear of children from the consequences of pain.
- Some children worry that they might have committed wrongful acts, errors, sins so that they must be inflicted with pains and sufferings. Pain for these children can be conceptualized in the sense of punishment or torture (Jongudomkarn, et al., 2006).
- Children do not choose pain or painful events; hence, suffering from pain is a compulsion for them (Meldrum, et al., 2009).

#### 3.4.5 Pain descriptors

In terms of describing pain, it should be noted that every child has a subjective description of pain depending on their personal experiences (Kortesluoma, Nikkonen, et al., 2008; Kortesluoma, Punamäki, et al., 2008). In some of the pain descriptors, the concept of pain can be extracted from children's perspectives:

1. Pain description in healthy children with limited experiences of pain or experiences in their normal life compared with sick children with different, unusual pain experiences can contain various aspects and priorities. Healthy children mostly consider the physical aspects of pain including causes of pain, but sick children may describe the emotional and psychological aspects of pain (Kortesluoma, Nikkonen, et al., 2008; Kortesluoma, Punamäki, et al., 2008; Stefanatou & Bowler, 1997).
2. The quality of perception and description of acute and chronic pain is different. The chronic pain in children is conceptualized as severe and persistent discomfort that cannot be cured and thus mental involvement in pain is never‐ending. What is certain is that children, like adults, see chronic pain as highly debilitating and affecting the whole course of life. Return to normal life is the most important desire in children, and in some cases, they are even ready to have pain but live like other children. Therefore, they focus on aspects that reflect their fatigue and their inability to deal with pain (Crofford, 2015; Tsao, Evans, Seidman, & Zeltzer, 2012). On the other hand, this concept of pain in the minds of children suggests an inadequate evaluation and control of chronic pain. Describing acute pain, children often consider pain as a permanent event and assume that it will kill them, due to their lack of prior experience of pain; however, over time, they discover that this pain is temporary and soothing (Cheng, Foster, & Hester, 2003; Cheng, Foster, & Huang, 2003; Crow, 1993; Degotardi, et al., 2006). For children with cognitive impairment, their pain experiences may go unnoticed due to their inability to distinctively describe or express themselves (RCN, 2009). Moreover, children live through negative experiences of hospitalization and treatments because of acute pain of a physical harm, and they seek for non‐pharmacological and non‐invasive methods of pain relief (Borghi, et al., 2014). Some pain is sometimes relieved without any need for special actions, and children may express their experiences of relaxation techniques such as watching TV especially cartoons or fondling (Neuman, 1996). Nevertheless, children suffering from chronic pain often feel reluctant to use any relaxation techniques.
3. From children's perspectives, the colours of red and black represent pain (Savedra, et al., 1982) and they paint the location of pain or the pain itself or the person suffering using these two colours in most of their drawings. Red indicates the climax of emotions, and children display their discomfort and pain with the help of this colour and separate it from other parts of the body (Jongudomkarn, et al., 2006; Unruh, et al., 1983). Additionally, use of red for pain is influenced by bleeding, inflammation and heat that children have experienced after injuries or wounds. So, red or pain means a physical injury or an uncomfortable feeling which is mainly a reflection of the external source of pain. The colour black also gives an unknown sense of ambiguity and sadness and shows the space smaller and similarly creates a sense of formality and obligation. Through choosing this colour, children can express the pain causing an obscure or uncomfortable feeling in them and sometimes they do not know the cause. Nevertheless, this pain hurts them and must be recognized and taken into consideration by others. Moreover, this colour refers mostly to the internal or emotional sources of pain. So, the colours of red and black for children are conceptualized as the pain they have experienced (Jongudomkarn, et al., 2006; Savedra, et al., 1982; Unruh, et al., 1983).
4. Children have the following characteristics in terms of expressing and describing pain:
  - They generally act in a self‐centred manner. That is, they speak of their own unique experiences of pain (Cheng, Foster, & Hester, 2003; Cheng, Foster, & Huang, 2003).
  - Some children do not define pain but analyse it as an object or an incident such as a finger cut or bodily harm (Meldrum, et al., 2009).
  - In describing pain, children do not see themselves as part of pain and they internalize it.
  - Children's statements and descriptions of pain are based on reality except for a few things that may be beneficial to their pain. There is almost always a real physical or emotional pain and they speak of a pain experienced in reality (Jongudomkarn, et al., 2006).

Given the remembrance or description of their pain, they always refer to it as bad, unpleasant and uncomfortable aspects. They like to be cuddled, receive attention, watch cartoons and enjoy what others can do for them when they suffer from pain. In some cases, such relief is so attractive to them that they are called pain benefits (Crow, 1993; Neuman, 1996).

### 3.5 Identify a model case

According to the concept of pain in children and its characteristics, two models are illustrated for chronic pain in a sick and hospitalized child and acute pain in a healthy child:

1. A child aged 6 years suffering from kidney failure is hospitalized and treated for a long time. The nurse is reviewing and recording the vital signs of the child. At the same time, the nurse notices one of the child's drawings is different from other ones. The child has drawn a hospital bed using three horizontal and vertical lines in the middle of a white page. There is also a black dot at the centre of the bed, and there is a larger spot in red on this dot (the colour of pain is one of the pain descriptors). The nurse asks questions from the child about the painting and the child replies: “This is my bed, this black dot is me. This red spot on me is my kidney that hurts. My kidney always hurts. I do not know where the pain comes from, but it is now in my tummy (pain has an internal source). The pain made me sick (pain is a physical harm). I do not like this pain because I am not ever happy and I am crying (pain is a negative feeling) and I have to stay in the hospital and suffer from shots (pain is a concern). Some days, my mom goes home and I feel lonely, this time my heart also hurts and I feel sad more and more (source of emotional pain). I wish this pain was away from my tummy. It seems that someone is in my tummy, constantly punching my kidney, never gets tired and never leaves my tummy” (use of analogy, reality‐based pain, self‐centred description of pain, attention to chronic and uncomfortable pain and unrelieved pain, more reference to emotional and psychological aspects of pain and uncomfortable aspects of pain are considered as pain descriptors). This model involves the implementation of the concept of chronic pain in a child who suffers from persistent pain due to chronic pain and considers oneself weak and delicate in coping with pain.
2. A 5‐year‐old child describes one's painful experience as follows: “I was playing soccer with my friends when I sprained my foot and hit the ground (external source of pain). I injured my knee. It bled and ached (pain is a physical harm). The pain was similar to pouring boiling water on my leg. I felt it burning and I cried (pain is a negative feeling). I was afraid my parents would take me to the doctor and give me a shot. I was not able to play with my friends because I had pain (pain is a concern). But my mom bandaged my leg, I slept and then my pain was relieved.” This child drew a picture of a child standing with a sad face. The child had shaded the middle part of one's left leg in black with a multiplication sign to mark plasters on it. This model involves the implementation of the concept of acute pain in a healthy child. Pain descriptors also include a self‐centred description based on objective reality, use of colour to represent pain, more attention to the causes and the physical aspects of pain, a reference to negative aspects of pain, attention to alleviation and temporary nature of pain and use of analogy.

### 3.6 Identify antecedents and consequences

#### 3.6.1 Antecedents

Children's experiences of pain are influenced by various factors associated with it. The presence of painful stimulus, the age of a child, stage of growth and development, prior experience of pain, fear and anxiety remained from previous painful experience, responses and reactions by others towards pain, psychological states of the child, acute and chronic pain, and cultural and social contexts wherein children are living are among the antecedents for the establishment of the concept of pain in children (Cheng, 2002; Gaffney & Dunne, 1986; IASP, 2005).

#### 3.6.2 Consequences

The concept of pain formed up in children can be reflected in various ways such as verbal expression or description of pain and behavioural symptoms such as avoiding painful situations, crying, drawing and storytelling. From children's perspectives, pain is considered as a bad event, and it should be relieved as quickly as possible. Therefore, the consequences of pain can lead to appropriate or inappropriate relief. Depending on the concept that children have gained from pain, they learn how to cope with their mild and acute pains or reduce stress. However, children need more help from others in the face of more severe and chronic pain. Otherwise, pain is not relieved correctly, and it can be accompanied by psychological effects. Generally, the concept of pain in children appears in different ways, and it can affect pain behaviour in children (Bienvenu, et al., 2011; Cheng, 2002; Cheng, Foster, & Hester, 2003; Cheng, Foster, & Huang, 2003; Jongudomkarn, et al., 2006; Meldrum, et al., 2009; Savedra, et al., 1982).

### 3.7 Define empirical referents

There are numerous pain measurement tools appropriate for children's age and their cognitive and language skills. Wong‐Baker Faces Pain Rating Scale, Faces Pain Scale‐Revised (FPS‐R) and Oucher Scale are common self‐reporting tools to measure the severity of pain in children (IASP, 2005). A body outline marking is a self‐reporting tool that evaluates the location of pain (Savedra, Tesler, Holzemer, Wilkie, & Ward, 1989). Child behavioural responses to pain can be evaluated by observational instruments such as The Observational Scale of Behavioral Distress (OSBD) and The Faces Legs Activity Cry Consolability Scale (FLACC) (Srouji, Ratnapalan, & Schneeweiss, 2010). In this respect, interviews using Children's Pain Perspectives Interview (CPPI) or interpretation of drawings of pain can be useful in examining perceptions and experiences of pain in children (Crow, 1993).

In summary, the concept of pain in children forms up with multiple antecedents related to child and pain characteristics and external factors. From children's perspectives, pain is a physical harm and a negative feeling associated with external, internal and emotional concerns. Their descriptions of pain also include indices such as the colour of pain, the analogy of pain and self‐centred pain description. So, the consequences of the concept of pain in children can have two dimensions. First, the concept of pain in children can be manifested in different ways; secondly, the concept of pain would have an impact on pain behaviour in children. Because of its subjective nature, pain itself is not directly measured; however, the effects of pain or methods by which pain appears are often evaluated. The nature of pain experienced by interviews and drawings is also measurable.

## 4 DISCUSSION

In this study, we attempted to clarify the concept of pain among children through a concept analysis. Children's descriptions and perceptions of pain were outlined. One of the major strengths of the current analysis is the identification of new aspects of the concept of pain in children and a clearer understanding and interpretations of drawings children use to describe the pain. We strongly believe this would guide clinicians in making meaningful interpretations of children's subjective pain experiences, hence leading to accurate diagnoses and treatment. Since there are several reports of hospitalized children still experiencing pain (Birnie, et al., 2014; Friedrichsdorf, et al., 2015; Harrison, et al., 2014; Taylor, Boyer, & Campbell, 2008), clinicians would be well placed to give appropriate pain management if children's pain descriptions and concerns and better understood. It should be noted that pain is a multidimensional phenomenon and concept that can be defined in various dimensions. No aspect by itself can give a complete definition of the concept of pain. However, for a complete definition of pain, a set of viewpoints towards the concept and the meaning of pain are required. Children's perspectives must be considered as essential parts in illustrating the complex concept of pain. Such concepts can be evident in children with the development of speech and language skills in. Thus, children can express their perceived concepts of pain experiences.

Analysing the concept of pain according to children's statements and descriptions, this study illustrated the nature and some characteristics of pain in children. There have been lots of different concept analyses about pain (Chang, Oh, Park, Kim, & Kil, 2011; Cheng, Foster, & Hester, 2003; Cheng, Foster, & Huang, 2003; Larner, 2014; Mahon, 1994; Montes‐Sandoval, 1999). However, in these analyses, the concept of pain has mostly been discussed in general terms and not specifically centred on children. Also, the characteristics of pain in children are often not considered and illustrated (AAP, 2001). Thus, this issue distinguishes the present study from other concept analyses in the related studies about pain.

Presenting the definition of acute pain in children, the American Academy of Pediatrics suggests that acute pain is the most common lived harm experienced by children because of injuries, illnesses or medical procedures (AAP, 2001). This is consistent with our findings which illustrated how children perceive pain. However, in this definition proposed by AAP, senses of pain are not specified.

Lived experiences of injuries are ambiguous, and their nature is unclear. To clarify these uncertainties, the results of this present study from children's perspectives showed that pain was both a physical harm and a negative feeling. The source of this painful experience could be external, internal and emotional. These findings are strongly backed by the results of a concept analysis conducted by Mahon (1994). Even though her study was not specifically centred on children's pain perceptions, however, it emerged that pain experiences could present in similar forms (Mahon, 1994).

The WHO and the IASP have also considered pain as a tissue damage that can lead to unpleasant sensory and emotional experiences (IASP, 2005; WHO, 2012). This definition was included in this current analysis; nevertheless, pain in children does not only refer to tissue damage, emotional and mental harm are both equally considered as pain from children's perspectives. Children are also likely to express their mental states of emotional events as pain because of their cognitive impairment. According to McCaffery (1979), as cited in (Azize, Humphreys, & Cattani, 2011), pain is whatever a person can experience and express.

Based on the current concept analysis, children are endowed with the ability to define, describe and locate pain. They also express emotions and describe and adopt strategies associated with pain relief; hence, they can be a comprehensive source of assessing their own pain. This again is consistent with the results of a study conducted on hospitalized‐based children in the United States. Thirty‐seven interviewed children were able to describe the source of their pain experiences—from procedural‐related pains to disease and surgical‐related pain (Shomaker, Dutton, & Mark, 2015). Also, their study found that children's descriptions of pain experience were mostly varied based on age, with older children having higher documented reports of pain than younger children. This aspect of their finding also correlates with results of this current analysis.

The findings of this present analysis could add new dimensions and serve as a rich source of relevant data to the development and concept of pain in children. For example, children's concerns about pain and how to understand their descriptions and perceptions of pain are usually important elements in the development of pain measuring scales and tools (Bai & Jiang, 2015; Chang, Versloot, Fashler, McCrystal, & Craig, 2015; Massaro, et al., 2014). However, it should be noted that children's concerns of pain do not have a stable nature. They may depend on age and stage of growth and development to resolve pain concerns and change descriptions from abstract forms to more concrete ones. In other words, the concept of pain in children is time‐dependent, except for physical harm and negative feelings that remain stable and are consistent with other definitions in relation to the meaning of pain. Children's concerns and descriptions of pain can change over time.

The use of drawings as a way of communication among children is not new and has proven its usefulness in social and clinical settings (Driessnack, 2005; Monsen, 2003). During pain assessments, these methods could be used along with corresponding interpretations identified in this study and similar scientific studies to serve as the basis for better pain management.

Given all these achievements, concept analysis has a temporary nature and it may raise new questions considering time, place and based on individuality, which can trigger other concept analyses in future with regard to similar topics.

This study was the first concept analysis of pain in children conducted based on the literature available. The new approach in the field of pain research is towards conducting studies on pain in children, but it still requires the acquisition of fundamental and applied knowledge in the field. Certainly, the development of further studies on pain in children and other concept analyses can lead to the identification of more aspects of the meaning and the concept of pain in children.

## 5 CONCLUSION

Although pain has previously been studied extensively, this current analysis opens another dimension to the perception of pain in children. Children at various stages of development perceive and describe pain differently. Their descriptions are influenced by what concerns them most about the pain they are feeling and whether their pain is acute or chronic. Children perceive pain as internal, external or emotional. When preparing pain evaluation tools, it is necessary to address the characteristics of pain. In the case of chronic pain, emotional effects of pain on children's psyche need extra attention. Child‐based pain management guidelines can then be formulated with the results of relevant concept analyses. Pain assessment is a major part of pain management in children. By considering the characteristics of the concept of pain, the efficiency and usefulness of the developed tools can be enhanced to create an advancement in paediatric pain management.

## 6 IMPLICATIONS FOR NURSING

This concept analysis adds to the already existing literature of pain in children. The results could serve as clinical acumen for doctors, nurses, other clinicians and theorists concerned with understating the concept of pain in children. Moreover, it could be used as an educational and a practical guide for students and would benefit parents and families who wish to understand children's perceptions of pain as well. One of the important tasks of nurses is to manage and control pain in children. Pain assessment and evaluation are also taken into account as an important part of pain management in children; thus, the efficiency and the usefulness of pain related tools can be improved through considering the characteristics of the concept of pain. This can lead to newer discoveries and effective pain management in children. For example, this approach can contribute to the development of different tools for patient pain assessment in healthy and sick children. Given that children know pain as a kind of concern, nurses can lessen the negative effects of pain through identification of these concerns and teach proper self‐control methods for fears and concerns in children and consequently assess and treat the nature of the pain in a better way. Clinicians involved in caring for children should consider the importance of assessing pain as the fifth vital sign and identify and treat pain appropriately by considering the components and the characteristics of the concept of pain that were illustrated in this study. However, further research will be required in specific contexts since the dynamism of pain sometimes renders global pain ratings and interpretations less effective. This will further support the growing need for understanding pain in children.

## 7 LIMITATIONS

The concept of pain is complex, even among adults. Therefore, we admit that this study is far from conclusive; hence, further studies are required to broaden the understanding of children's perceptions and descriptions of their pain experiences. This analysis relied on the currently available evidence and was constrained in finding specific cultural and context‐based texts; therefore, the results were representative of a broader concept of pain in children, though with emphasis on descriptions of pain using drawings and dialogs. For this reason and for the fact that in most cases interpretations were subjective reports of children's pain experiences, we caution readers to be mindful of generalizing the results.
